# Advanced CNN models in gastric cancer diagnosis: enhancing endoscopic image analysis with deep transfer learning

**DOI:** 10.3389/fonc.2024.1431912

**Published:** 2024-09-16

**Authors:** Priya Bhardwaj, SeongKi Kim, Apeksha Koul, Yogesh Kumar, Ankur Changela, Jana Shafi, Muhammad Fazal Ijaz

**Affiliations:** ^1^ Department of Computer Science and Engineering (CSE), Tula’s Institute, Dehradun, India; ^2^ Department of Computer Engineering, Chosun University, Gwangju, Republic of Korea; ^3^ School of Computer Science Engineering and Technology, Bennett University, Greater Noida, India; ^4^ Department of Computer Science and Engineering (CSE), School of Technology, Pandit Deendayal Energy University, Gandhinagar, India; ^5^ Department of Information and Communication Technology (ICT), School of Technology, Pandit Deendayal Energy University, Gandhinagar, India; ^6^ Department of Computer Engineering and Information, College of Engineering in Wadi Alddawasir, Prince Sattam Bin Abdulaziz University, Wadi Alddawasir, Saudi Arabia; ^7^ School of Information Technology (IT) and Engineering, Melbourne Institute of Technology, Melbourne, VIC, Australia

**Keywords:** gastric cancer, medical images, deep learning, ulcerative colitis, transfer learning, contour features

## Abstract

**Introduction:**

The rapid advancement of science and technology has significantly expanded the capabilities of artificial intelligence, enhancing diagnostic accuracy for gastric cancer.

**Methods:**

This research aims to utilize endoscopic images to identify various gastric disorders using an advanced Convolutional Neural Network (CNN) model. The Kvasir dataset, comprising images of normal Z-line, normal pylorus, ulcerative colitis, stool, and polyps, was used. Images were pre-processed and graphically analyzed to understand pixel intensity patterns, followed by feature extraction using adaptive thresholding and contour analysis for morphological values. Five deep transfer learning models—NASNetMobile, EfficientNetB5, EfficientNetB6, InceptionV3, DenseNet169—and a hybrid model combining EfficientNetB6 and DenseNet169 were evaluated using various performance metrics.

**Results & discussion:**

For the complete images of gastric cancer, EfficientNetB6 computed the top performance with 99.88% accuracy on a loss of 0.049. Additionally, InceptionV3 achieved the highest testing accuracy of 97.94% for detecting normal pylorus, while EfficientNetB6 excelled in detecting ulcerative colitis and normal Z-line with accuracies of 98.8% and 97.85%, respectively. EfficientNetB5 performed best for polyps and stool with accuracies of 98.40% and 96.86%, respectively.The study demonstrates that deep transfer learning techniques can effectively predict and classify different types of gastric cancer at early stages, aiding experts in diagnosis and detection.

## 1 Introduction

**Figure 1:**
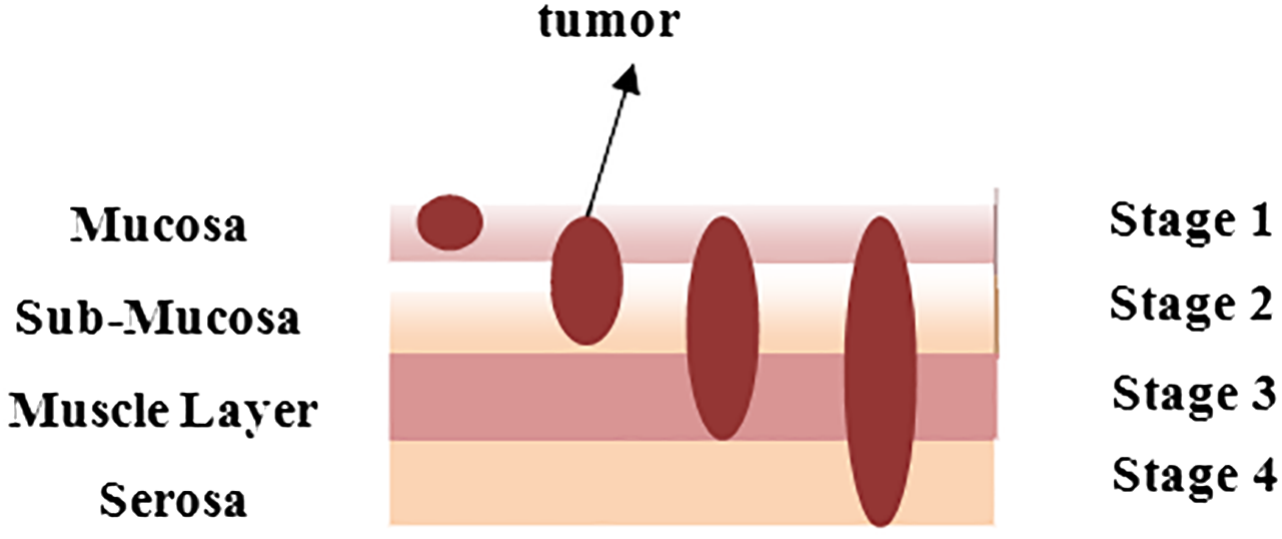
An overlay to indicate the stage of tumor in stomach at different layers.

Gastric cancer, often known as stomach cancer, is the fifth most frequent cancer worldwide. Approximately 95% of the time, this sort of cancer begins in the stomach’s inner lining and subsequently grows and develops deeper into the stomach walls. It generally starts at the gastroesophageal junction, where the long tube that transports the food, you swallow meets the stomach. Cancer does not just affect the stomach; it also affects neighboring organs such as the liver and pancreas. **Figure 1** depicts the layered image of gastric cancer developing in a human stomach from stage 1 to stage 4 (1).

Nonetheless, a few demographic factors, such as age above 65, male gender, and ethnicity from East Asia, South or Central America, or Eastern Europe, can increase the risk (2). In the early stages, people with gastric cancer frequently report indigestion and stomach discomfort, mild nausea, heartburn, a loss of appetite, blood in the stool, vomiting, abrupt weight loss, jaundice, difficulty swallowing, and ascites (3). In 2022, the American Cancer Society predicts that around 26,380 new instances of stomach cancer will be documented, with 15,900 males and 10,480 females. In comparison, 11090 people have died thus far, including 6,690 men and 4,400 women (4). With the devastating impact of stomach cancer on people in mind, doctors or oncologists have recommended a variety of treatments to treat the cancer and preserve people’s lives. **Table 1** summarizes the clinical procedures used to prevent the formation of stomach cancer cells (5, 6).

**Table 1:** Traditional ways to diagnose gastric diseases.

Treatments	Description
**Health history of patients**	An examination of the body which includes symptoms of health, the appearance of lumps etc.
**Complete blood count (CBC)**	The quantity of white blood cells, red blood cells, and platelets are counted including amount of hemoglobin in the RBCs.
**Upper endoscopy**	It is a thin, illuminated tube used to inspect the esophagus, stomach, and duodenum (initial section of the small intestine) for abnormalities.
**Barium swallow**	The patient is given liquid containing barium (a silver-white metallic substance) that coats the esophagus and stomach, and x-rays are taken.
**CT scan**	A computer coupled to an x-ray machine creates the images in this method. To make the organs or tissues more visible, a dye may be ingested or injected into a vein.
**Biopsy**	In this the cells or tissues are removed and examined for signs of malignancy under powerful microscope.

Overall, gastric or stomach cancer was generally identified at advanced stages due to its hidden and similar symptoms, which resulted in a miserable diagnosis. Early correct identification of gastric cancer has been observed to raise the overall 5-year survival rate by approximately 90% (4). However, the number of experienced imaging experts limits early stomach cancer diagnosis. Furthermore, diagnosis accuracy was highly dependent on expert clinical expertise and was susceptible to various circumstances (7).

Then came the AI era, during which artificial intelligence (AI) got great attention in various medical sectors. AI approaches, which used a computer to mimic human cognitive function, were applied to process and evaluate vast volumes of data and might aid gastroenterologists during clinical diagnosis and decision-making (8). In fact, the relevance of AI in cancer research and therapeutic application is becoming well-recognized. Cancers such as gastric or stomach cancer are suitable to test for determining whether early efforts to apply AI to provide medicine to patients will deliver valuable outcomes or not as researchers have used AI to help diagnose specific endoscopic tests (9).

The proposed study leverages advanced deep learning models to enhance the detection and classification of various stomach conditions, marking significant contributions to the field of medical imaging and gastrointestinal disease diagnosis. The key contributions and novelty of the work can be outlined as follows:

- Usage of a large and diverse set of images from the KVASIR collection which encompasses multiple conditions such as normal pylorus, polyps, Ulcerative Colitis, normal z-line, and stool.
- Pre-processing of images which include the creation of RGB histograms and extraction of regions of interest, is crucial for improving model performance. The detailed graphical representation and contour feature extraction help in accurately identifying the relevant features for each condition, which is essential for the training of deep learning models.
- Applying multiple state-of-the-art deep transfer learning models such as NASNetMobile, Inception V3, EfficientNetB5, EfficientNetB6, and DenseNet169 allows for a comprehensive comparison of their performance.
- The integration of EfficientNetB6 and DenseNet169 into a hybrid model represents a novel approach. Combining these models leverages the strengths of both architectures, potentially leading to improved performance in terms of accuracy, precision, recall, and F1 score. The applied novel model has been rigorously compared with existing techniques using both the same dataset and a different dataset of gastric cancer. The comparative analysis demonstrates that the hybrid approach offers a more robust solution for detecting and classifying gastric cancer.
- The findings from this study provide a valuable benchmark for future research in the field of medical imaging and AI-based diagnosis of gastric cancer. The methodologies and results can guide subsequent studies, helping to refine and improve deep learning models for similar applications.

The following section presents the research conducted in the field of detecting gastric cancer is discussed; the methodology to conduct the research which is used to classify gastric cancer is presented in section 3 whereas results and discussion is covered in the section 4, and the Section 5 summarizes the paper with challenges and its scope in future.

## 2 Literature work

Various researchers have worked to diagnose gastric cancer using multiple artificial intelligence techniques.

Additionally, the work of the researchers has been also analyzed and the challenges which they had faced are also pointed out in **Table 2**.

**Table 2:** Analysis of Previous work of the researchers to detect and classify gastric diseases.

Author’s Name	Dataset	Techniques	Outcome	Limitations
Li et al. (2018) (12)	560 gastric slices, 140 normal slices	Gastric Net	Average classification accuracy = 97.93%	Limited dataset
Nadeem et al. (2018) (10)	MediaEval 8000 images	VGG-19, logistic regression	Accuracy= 83%	Low accuracy
Hirasawa et al. (2018) (13)	13584 endoscopic images	CNN	Sensitivity = 92.2% Positive predicted value = 30.6%	The model was trained with the high quality images which means it won’t work for post-biopsy bleeding, images and less insufflation of air.
Song et al. (2020) (14)	PLAGH dataset	DeepLabV3	Average specificity = 80.6%	The cost of computation was high
Yuan & Meng (2017) (20)	WCE image dataset	stacked sparse autoencoder	Accuracy= 98%	Needs improvement in classification accuracy
Mortezagholi et al. (2019) (15)	Samples of 405 patients	KNN, Naïve Bayes, SVM	Accuracy = 90.8% F1 score = 91.99%	class imbalance
Aslam et al. (2020) (16)	Data collected from 220 samples of cancerous and non-cancerous stomach	Support vector machine, linear kernel	Accuracy = 97.18% Specificity = 97.44% F1 score = 91.99%	SVM failed to predict 14 instances
Ueyama et al. (2021) (17)	Dataset of 5574 magnifying narrow brand imaging	Deep learning, CAD system	Accuracy = 98.77% Specificity = 100% Sensitivity = 98%	Sometime it was difficult for the model to distinguish from gastritis.
Zhou et al. (2014) (19)	359 frames of video capsule endoscopy	Support vector machine classifier	Accuracy= 90.77%	Some algorithms must be incorporated for the robustness of the approach
Liu et al. (2019) (18)	557 patients of gastric cancer	Support vector machine, autoencoder, logistic regression	Accuracy = 89% Specificity = 79% Sensitivity = 78% F1 Score = 95%	Small dataset
Asperti & Mastronardo (2017) (22)	Kvasir dataset	Inception	Accuracy=91.55% Precision = 91.5%	The system was required to diagnose other GI tract-based disorders.
Liu et al. (2018) (21)	Dataset of Gastric pathology images	Artificial neural network	F-score = 0.96	This approach needs further improvement of classification accuracy
Sun et al. (2020) (24)	Dataset of annotated gastricscopic images	Deep neural network	Accuracy = 96.7% Recall = 94.9% F1score = 94.7%	Overfitting

*Nadeem* et al. *(2018)* (10) introduced a innovative ensemble method that integrated texture features as well as deep learning features to enhance the prediction of abnormalities in gastrointestinal (GI) tract, such as Peptic Ulcer Disease. For extracting data from visual information, they used multimedia content analysis and for classification, machine learning techniques had been applied. On combining these two approaches, the ensemble method improved the accuracy as well as effectiveness to detect GI tract abnormalities. *Chen* et al. *(2022)* (11) developed a multi-scale visual transformer model also named as GasHis-Transformer to automatically detect gastric cancer in histopathological images collected from gastric histopathologic image dataset. They also used a Dropconnect-based lightweight network to facilitate clinical application as well as reduce the model size and training time to maintain high confidence levels. *Li* et al. *(2018)* (12) proposed a deep learning-based framework called GastricNet to automatically detect gastric cancer. GastricNet employed different architectures for its shallow and deep layers to enhance feature extraction. The performance of their framework was examined on using publically available BOT gastric slice dataset. *Hirasawa* et al. *(2018)* (13) focused on applying artificial intelligence and deep learning, specially through convolutional neural networks, to enhance recognition of image in medical diagnostic imaging. The CNN-based totally diagnostic system was developed using Single Shot MultiBox Detector structure and trained with 13,584 endoscopic snap shots of gastric cancer. Later to evaluate its diagnostic accuracy, the overall performance of CNN model was tested on an impartial set of 2,296 stomach snap shots accrued. *Song* et al. *(2020)* (14) aimed to develop a clinically applicable artificial intelligence system to early identify and diagnose histopathological images of gastric based cancers. Their goal became to ease the workload of pathologists and boom diagnostic accuracy with the aid of using a deep convolutional neural community trained on pixel-degree annotated H&E-stained entire slide images. The system turned out to be robustly in real-time data, and suggested its feasibility and benefits for routine exercise in clinical settings. *Mortezagholi* et al. *(2019)* (15) investigated the effectiveness of diverse traits of disease risk aspect and data mining techniques to expect and diagnose gastric most cancers. Specifically, they aimed to determine how properly distinct machine learning models including Support Vector Machine, Decision Tree, Naive Bayesian Model, as well as k-Nearest Neighbors may be used for the type of people having gastric cancers or being healthful, based on a fixed of eleven characteristics and risk elements. *Aslam* et al. *(2020)* (16) developed an advanced classification and prediction system for diagnosing gastric cancer (GC) through the analysis of saliva samples. By focusing on the early detection of gastric cancer (EGC), the study aimed to significantly improve patient survival rates. Utilizing high-performance liquid chromatography-mass spectrometry (HPLC-MS), the researchers identified fourteen amino acid biomarkers in saliva samples that could distinguish between malignant and benign conditions. The study employed support vector machine (SVM) models with various kernels for binary classification, utilizing a processed Raman dataset for training and testing. *Ueyama* et al. *(2021)* (17) developed and evaluate an artificial intelligence (AI)-assisted computer-aided diagnosis (CAD) system utilizing a convolutional neural network (CNN) to enhance the diagnosis of early gastric cancer (EGC) through magnifying endoscopy with narrow-band imaging (ME-NBI). Given the substantial expertise required for accurate ME-NBI diagnosis, the study aimed to leverage deep learning, specifically the ResNet50 model, to create a reliable diagnostic tool. *Sun* et al. *(2020)* (24) designed and evaluated a machine learning-based clinical decision-support model to predict the extent of lymphadenectomy (D1 *vs*. D2) required for patients with locally advanced gastric cancer (GC). This aimed to address the ongoing controversy regarding the optimal surgical resection strategy for potentially curable GC and to limit unnecessary surgical treatments. Utilizing clinicoradiologic features from routine clinical assessments of 557 patients who underwent standard D2 resection, the study retrospectively interpreted these features with a blinded expert panel. The decision models developed using logistic regression, support vector machine, and auto-encoder algorithms, were trained on 371 cases and tested on 186 cases. In their study, *Yong* et al. *(2023)* (36) improved the ability to detect stomach cancer at an early stage by creating ensemble models. These models aggregated the decisions of numerous deep learning models to aid pathologists in analyzing histopathological images. The researchers suggested combining the results of multiple deep learning models to create ensemble models. The efficacy of these proposed models was assessed using the publicly accessible Gastric Histopathology Sub-size Image Database. The experimental results showed that the top 5 ensemble models obtained the highest level of detection accuracy among all sub-databases, with the most accurate model obtaining a detection accuracy of 99.20% in the 160 × 160 pixels sub-database.

## 3 Methodology

**Figure 2:**
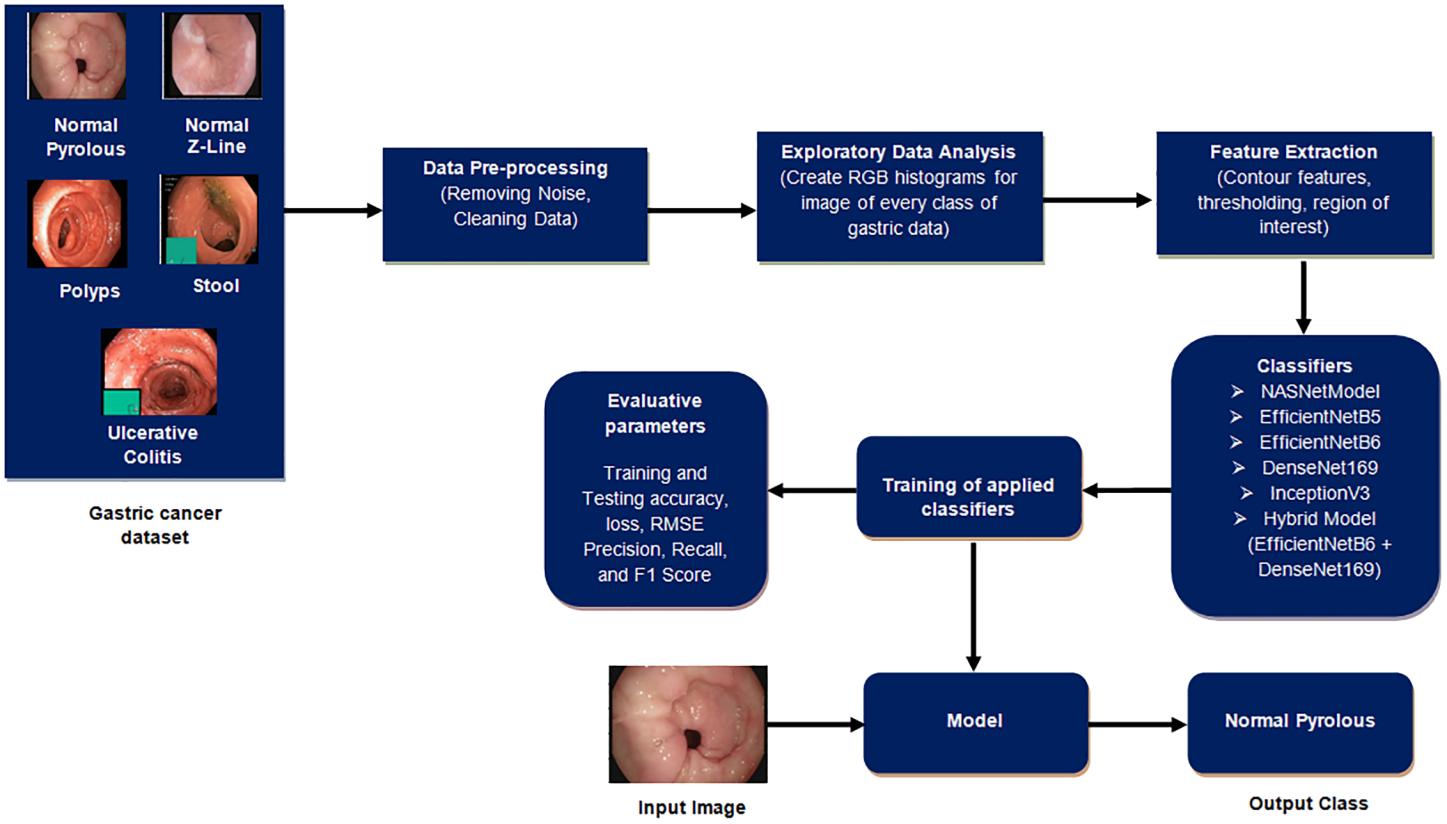
Proposed gastric disease detection system.

This section outlines the procedural steps, as shown in **Figure 2**, for detecting gastric cancer in stomach medical imaging. The methodologies involve a structured approach, starting with data pre-processing to improve quality, and then conducting exploratory data analysis to gain insights into the dataset’s characteristics. Afterwards, feature extraction is used to extract important information that is essential for the detection task. The data is divided into training and testing sets to ensure effective model training and evaluation. Classifiers, crucial for identifying cancerous patterns, are then utilized followed by a thorough performance evaluation is carried out to measure the effectiveness of the methodologies used.

### 3.1 Implementation details

The work was done in Python utilizing several libraries, such as Tensor flow, Keras, as well as Imutlis to perform fundamental image processing operations such as skeletonization, rotation, translation, scaling, identifying edges along with the sorting of contours. Python’s OS module is used to create, modify, identify, and remove directories. Matplotlib, a Python data visualization and graphics library, are also used. One of the essential advantages of visualization is the ability to see vast volumes of data in simple formats. Seaborn is used for exploratory data analysis that is merged with matplotlib and pandas library. In addition, Scikit-learn, NumPy, as well as the OpenCV package are also used (20–23).

### 3.2 Dataset

The Kvasir dataset, collected at the Vestre Viken Health Trust (VV) in Norway, encompasses a comprehensive collection of images acquired from endoscopic procedures. VV consists of four hospitals i.e. the Bærum Hospital, which houses a prominent gastroenterology department and contributes significantly to the dataset. The images are meticulously annotated by experienced medical experts from both the Cancer Registry of Norway (CRN) and VV, ensuring high-quality labels. CRN, associated with Oslo University Hospital Trust, focuses on cancer research and national cancer screening programs. The dataset includes hundreds of images for various classes, encompassing anatomical landmarks such as the Z-line, pylorus, and cecum, as well as pathological findings like esophagitis, polyps, and ulcerative colitis. The images come in different resolutions, ranging from 720x576 to 1920x1072 pixels, and are organized into separate folders based on their content. Some images feature a green picture-in-picture overlay illustrating the position and configuration of the endoscope within the bowel, using an electromagnetic imaging system. This additional information can support image interpretation but requires careful handling for detecting endoscopic findings.

**Figure 3:**
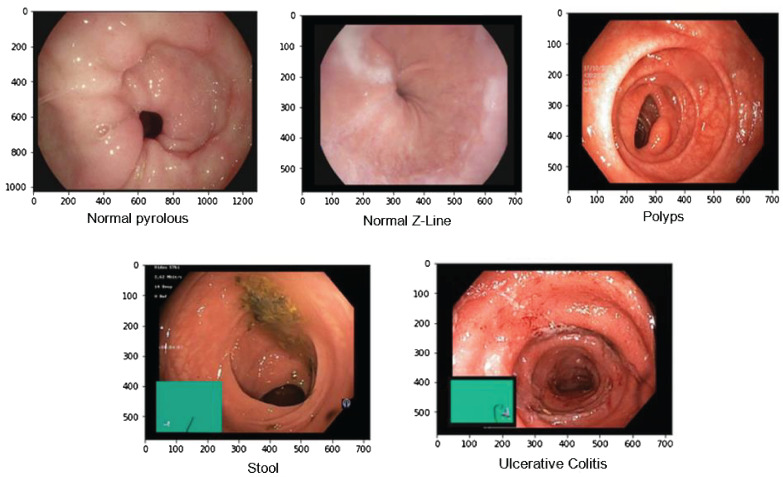
Samples of gastric diseases taken from Kvasir dataset.

Overall, the Kvasir dataset is highly diverse, with a sufficient number of images to support a wide range of applications which includes retrieving of image retrieval, machine and deep learning along with the transfer learning. For this paper, we took images of only a few diseases which include 4100 images of Normal Pylorus, 5000 images of Normal Z line, 4100 images of polyps, 5000 images of stool, and 5091 images of Ulcerative Colitis. **Figure 3** shows the sample of images taken from the dataset (25).

### 3.3 Pre-Processing for endoscopic images

In the pre-processing stage of image data, a critical step is undertaken prior to any classification techniques, particularly in the context of a dataset containing images of gastric diseases (24). Initially, the images are loaded from the dataset into the system. This is facilitated by the `Opencv_window` method, which creates a new window with a specified name and flag. This window allows the images to be displayed on the screen, enabling the user to visually analyze and comprehend the data. As mentioned in the description of the dataset, the images are saved in various resolutions, which can affect the performance of the classification model. Therefore, resizing the images to a uniform resolution is crucial. OpenCV provides functions like `cv2.resize()` to resize the images to a standard size, ensuring consistency across the dataset. Later, to standardize the pixel values across the images, normalization is performed. This involves scaling the pixel values to a specific range, usually [0, 1] or [-1, 1], to facilitate faster convergence during model training. OpenCV can be used to normalize the images by dividing the pixel values by the maximum pixel value (usually 255 for 8-bit images). In addition to this, when dealing with large datasets, managing multiple images efficiently is crucial. The Imultis library aids in handling and organizing batches of images for streamlined processing. This includes loading images in batches, applying pre-processing steps, and preparing the data for model training.

### 3.4 Exploratory data analysis

**Figure 4:**
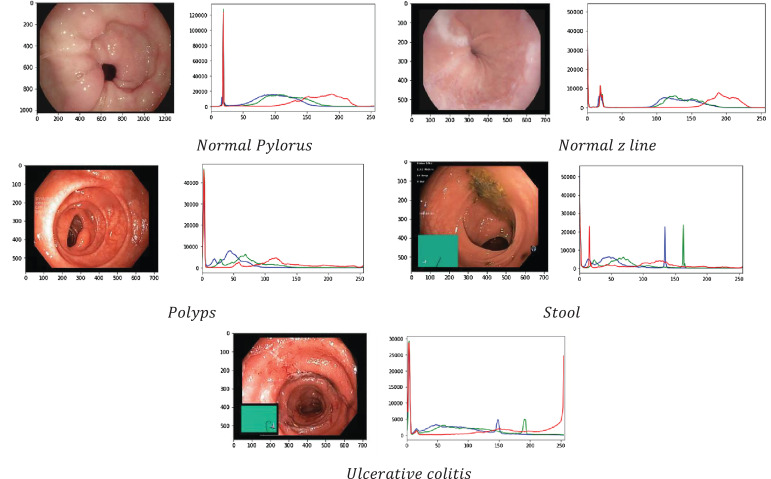
RGB Histogram of medical images of gastric cancer.

This section displays the information of the image graphically in the form of RGB histograms where R stands for Red, G stands for Green, and B stands for Blue. These histograms are useful to show how often different pixel intensities appear in an image for every single color channel. **Figure 4** shows the dispersion of image intensity, which is a number that illustrates how many pixels are used to show how intense each value is. This information has been used to figure out the image’s contrast and brightness. In fact, histogram equalization is used to map the distribution of one intensity to another so that the spread of intensities can be improved. This is also useful to show details of the image that were hidden in areas with low contrast.

### 3.5 Feature extraction

In this section, the main focus is on segmenting the images in order to extract the region of interest and generate rectangular boundary boxes. The process begins by generating contour features, which serve as the basis for obtaining morphological values of the images. These values are obtained by calculating various parameters such as aspect ratio, width, epsilon, perimeter, equivalent, height, extent, area, and others, as detailed in Equations 1–17, presented in **Table 3**. These parameters are useful as they play an important role in characterizing the morphological aspects of the regions that have been segmented. While computing the values of these parameters, it provides us the information related to the structural properties of the images which enables us to understand the region of interest of an image deeply.

**Table 3:** Morphological information of images.

Parameters	Normal Pylorus	Normal Z line	Polyps	Stool	Ulcerative Colitis
Area	0.0	2.0	0.0	1.0	2.0
Perimeter	0.0	5.65	0.0	2.0	5.65
Epsilon	0.0	0.56	0.0	0.2	0.56
Width	1	3	1	2	3
Height	1	3	1	1	3
Aspect Ratio	1.0	1.0	1.0	2.0	1.0
Extent	0.0	0.22	0.0	0.11	0.22
Equivalent Diameter	0.0	1.59	0.0	0.59	1.59
Minimum Value	128.0	127.0	129.0	136.0	123.0
Maximum Value	128.0	144.0	129.0	137.0	138.0
Minimum Value Location	585,1012	583,551	527,559	147,553	272,552
Maximum Value Location	585,1012	584,551	527,559	148,553	271,552
Mean Color/ Intensity	128.0	134.6	129.0	136.5	132.6
Extreme Leftmost Point	585,1012	582,551	527,559	147,553	271,552
Extreme Rightmost Point	585,1012	584,551	527,559	148,553	273,552
Extreme Topmost Point	585,1012	583,550	527,559	147,553	272,551
Extreme Bottommost Point	585,1012	583,552	527,559	147,553	272,553


(1) area=height*width

(2) $$perimeter=\;\sqrt{({\left(x_{2}-x_{1}\right)}^{2}+{\left(y_{2}-y_{1}\;\right)}^{2}}$$

(3) epsilon=0.1*cv2*arclength (cnt,True)

(4) width=cv2.boundingRect(cnt)

(5) height=cv2.boundingRect(cnt)

(6) $$Aspect\;Ratio=\;\frac{width}{height}$$

(7) $$Extent=\;\frac{object\;area}{bounding\;rectangle\;area}$$

(8) $$Equivalent\;diameter=\;\sqrt{\frac{4*contour\;area}{\pi }}$$

(9) Minimum value=cv2.min()

(10) Maximum value=cv2.max()

(11) Minimum value Location=cv2.minMaxLo()

(12) Maximum value Location=cv2.minMaxLo()

(13) Mean Color=cv2.mean()

(14) Extreme Leftmost point=tuple(cnt(cnt[:,:,0].argmin()[0])

(15) Extreme Rightmost point=tuple(cnt(cnt[:,:,0].argmin()[0])

(16) Extreme Topmost point=tuple(cnt(cnt[:,:,0].argmin()[0])

(17) $$\begin{array}{c} Extreme\;Bottommost\;point\\ =tuple(cnt(cnt\left[:,:,0\right].argmin()\left[0\right]) \end{array}$$

**Figure 5:**
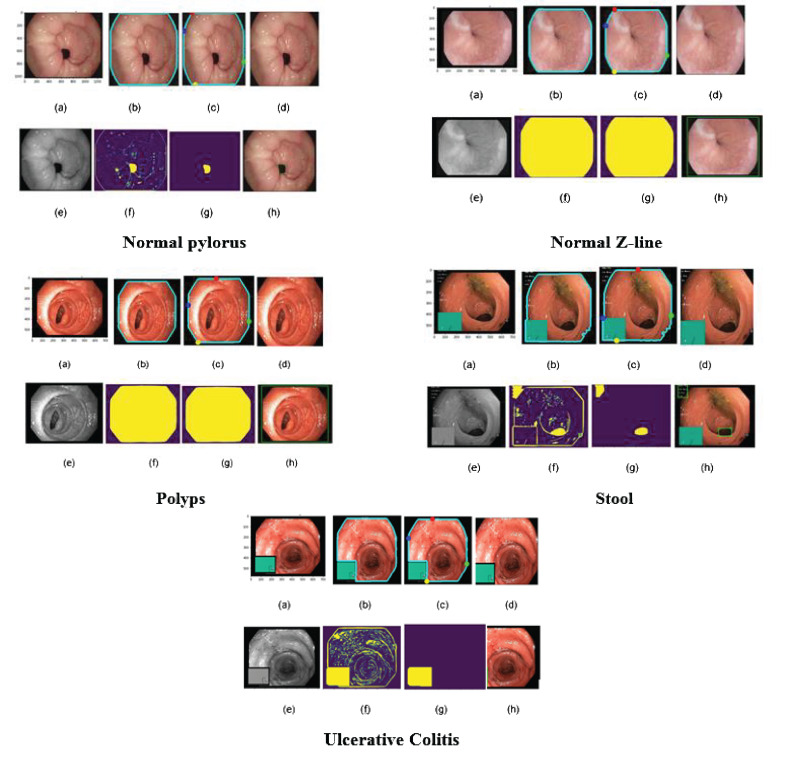
Feature extraction in endoscopic images. **(A)** colored image; **(B)** biggest contour; **(C)** extreme points; **(D)** cropped image; **(E)** grayscale image; **(F)** adaptive threholding; **(G)** morphological operation; **(H)** extracting ROI.

After computing the various parameters of the image to understand its characteristics, the biggest contour that covers the largest portion of the object of interest is determined. In addition to this, the image is cropped after the extreme points of the contour are generated in a continuous fashion. Later, the cropped image is converted to grayscale, which is done for simplifying the image by reducing it to a single channel and thereby facilitates subsequent processing. By comparing the pixel intensity values of each region in the image, the adaptive thresholding technique is used to distinguish the foreground objects from the background. The adaptive thresholding can be expressed as shown in Equation 18:


(18) T(x,y)=k*m(x,y)

where T(x, y) represents threshold value for the pixel at location (x, y), k means user-defined constant, and m(x, y) defines mean or median pixel intensity value of a local neighborhood which surrounds the pixel at location (x, y). Later, Morphologyex() is applied to the resultant image for extracting the morphological gradient of the foreground object. The morphological gradient is the difference between the dilation and erosion of the image. On the other hand, dilation involves adding pixels to the object boundary, which increases the size of the object. By computing the morphological gradient, the edge of the object is detected, and the region of interest is outlined. Overall, this process of morphological analysis and processing helps to isolate the region of interest from the rest of the image, making it easier to analyze and diagnose any gastric disease present in the image. All the results are shown in **Figure 5**.

### 3.6 Applied deep learning models

After extracting the features and splitting them in training as well as testing dataset, various pre trained learning models such as NASNetMobile, EfficientNetB5 and EfficientNetB6, DenseNet169, InceptionV3, and hybrid model (EfficientNetB6 and DenseNet169) have been used to get trained by both training as well as testing dataset. Apart from this, **Table 4** defines the chosen parameters used for the applied models for gastric cancer detection and classification.

**Table 4:** Hyper-parameters of applied deep learning models.

Parameters	Value
Learning Rate	0.001
Batch Size	32
Epochs	25
Optimizer	Adam
Loss function	Categorical cross entropy
Dropout rate	0.5
Weight decay	0.0001
Activation	Softmax
Rotation_range	30
Width_shift_range	0.1
Height_shift_range	0.1
Shear_range	0.2
Zoom_range	0.2
Horizontal_flip	True
Vertical_flip	False

#### 3.6.1 NASNet Mobile

It stands for Neural Architecture Search Network whose architectural design consists of two pivotal components: normal cells and reduction cells. The normal cells play a crucial role in determining the size of the feature map, shaping the fundamental characteristics of the network. On the other hand, reduction cells are responsible to capture relevant features by producing a feature map which is reduced by a factor of two in breadth as well as height as well as manages the complexity of the computational process.

The NASNet architecture works initially on a smaller dataset and later transfer learning technique is applied where its parameters are fine tuned to make it effectively work on the larger dataset (26).

Additionally, the NASNet Mobile architecture incorporates a control mechanism which is based on a Recurrent Neural Network (RNN). This RNN is uniquely used to predict the complete network structure. Leveraging two initial hidden states, the RNN also provides an adaptive as well as dynamic approach that allows NASNet Mobile for adjusting its architecture on the basis of learned patterns and information from the data. This not only enhances the prediction accuracy of NASNetMobile but also showcases its adaptability for diverse datasets which makes it a robust choice for various applications. The general structure of NASNetMobile can be represented by the following equation:


(19) $$\begin{array}{c} NASNETMobile\left(Input\right)\\ =\;Stem\left(Input\right)+\;\sum_{i=1}^{N}Cell_{i}\left(Input_{i-1}\right) \end{array}$$

Here, each $Cell_{i}$ represents a cell in the neural network, and *Stem* is the initial stem of the network that processes the input. The symbol Σ denotes the summation over all cells, and *N* is the total number of cells in the architecture. **Table 5** shows the layered architecture of NASNetMobile where the output shape of each layer is given along with its parameters.

**Table 5:** Layered architecture of NASNetMobile.

Layers	Output Shape	Parameters
**NASNetMobile** **Globalaveragepooling2d** **Dense layer** Batch normalization Activation function Dropout Dense layer	(None,6,6,1920) (None,1920) (None,256) (None,256) (None,256) (None,256) (None,5)	15921984 0 491520 1024 0 0 1285

As shown in **Table 5**, The NASNetMobile architecture produces an output shape of (None, 6, 6, 1920) and includes 15,921,984 parameters, this architecture leverages the efficiency of NASNet for feature extraction. Following this, a Global Average Pooling 2D layer reduces the dimensionality to (None, 1920) without adding any parameters, thus summarizing the spatial information. Next, a dense layer with 256 units is introduced, involving 491,520 parameters, followed by batch normalization, which introduces an additional 1,024 parameters. This batch normalization layer helps in stabilizing and accelerating the training process by normalizing the inputs. The subsequent activation function layer applies a non-linear transformation to enhance the model’s learning capability, though it does not add any parameters. To prevent overfitting, a dropout layer is utilized, which randomly sets a fraction of input units to zero during training. Finally, the architecture concludes with a dense output layer comprising 5 units, corresponding to the number of classes in the classification task. This layer includes 1,285 parameters.

#### 3.6.2 Efficient Network

EfficientNet has a compound coefficient for scaling the dimensions of both width and depth within neural networks. This unique approach has been designed for enhancing the accuracy as well as improvises the performance efficiency of model by reducing the number of parameters and Floating Point Operations Per Second (FLOPS) effectively.

The models of the EfficientNet are structured in such a way so that they can handle float tensors of pixels with values in the [0-255] range as inputs. This characteristic makes them compatible and versatile with standard representations of the image data. The simplified representation of EfficientNet is depicted in the following Equations 20–22:


(20) $$Width_{new}=\;Width_{old}\;\text{x }\emptyset^{\alpha }$$

(21) $$Depth_{new}=\;Depth_{old}\;\text{x }\emptyset^{\beta }$$

(22) $$Resolution_{new}=\;Resolution_{old}\;\text{x }\emptyset^{\gamma }$$

∅ = scaling vector, α ,  β , and γ = coefficients for determining how much aspect should be scaled. In the specific research, two variants of EfficientNet named as EfficientNet B5 and EfficientNet B6 are being used. The selection of these particular versions reflects a thoughtful balance between computational efficiency and model accuracy to indicate the intent of the research for achieving the optimal performance while conserving computational resources.

EfficientNet B5 and EfficientNet B6 are part of the EfficientNet model family, which effectively scales the dimensions of the network. This scaling enables the models for striking a balance between width, depth, as well as resolution to provide a versatile framework for handling multiple datasets and tasks.

In fact, EfficientNet B5 and EfficientNet B6 differ primarily in their scale, as EfficientNet B6 is larger and more complex than EfficientNet B5. This increased scale allows for a higher capacity to capture intricate or complex patterns and nuances within the data. The layered architecture of these models, as outlined in **Table 6**, provides a systematic representation of how various layers in the EfficientNet are organized (27).

**Table 6:** Layered architecture of EfficientNetB5 (left) and EfficientNetB6 (right).

EfficientNetB5			EfficientNetB6		
**EfficientNetB5**	(None,7,7,1792)	17268823	**EfficientNetB6**	(None,7,7,2048)	20539880
**Globalaveragepooling2d**	(None,1792)	0	**Globalaveragepooling2d**	(None,2048)	0
Dense layer	(None,256)	458652	Dense layer	(None,256)	5598288
Batch normalization	(None,256)	1024	Batch normalization	(None,256)	1024
Activation function	(None,256)	0	Activation function	(None,256)	0
Dropout	(None,256)	0	Dropout	(None,256)	0
Dense layer	(None,5)	1285	**Dense layer**	(None,5)	1285

The EfficientNetB5 model begins with a base EfficientNetB5 layer, outputting a tensor with the shape (None, 7, 7, 1792) and consisting of 17,268,823 parameters. This layer effectively captures and processes complex features from the input data. Following this, a Global Average Pooling 2D layer compresses the spatial dimensions, resulting in an output shape of (None, 1792) without adding any additional parameters. The subsequent dense layer, with 256 units, introduces 458,652 parameters, enabling the network to learn intricate patterns. Batch normalization is then applied, adding 1,024 parameters to standardize the inputs and improve training stability. An activation function layer follows, applying a non-linear transformation to the data but not increasing the parameter count. To mitigate overfitting, a dropout layer is included, which randomly drops a fraction of the input units during training. Finally, a dense output layer with 5 units, corresponding to the number of target classes, adds 1,285 parameters, culminating in a model designed for robust classification tasks.

Likewise, the EfficientNetB6 model follows a similar structure but starts with a more extensive base layer, producing an output shape of (None, 7, 7, 2048) and comprising 20,539,880 parameters. This larger base model allows for more detailed feature extraction and improved performance on complex tasks. A Global Average Pooling 2D layer then reduces the spatial dimensions to (None, 2048) without increasing the parameter count. The dense layer with 256 units is more parameter-heavy compared to EfficientNetB5, containing 5,598,288 parameters, which enhances the model’s learning capacity. Batch normalization, adding 1,024 parameters, follows to ensure consistent training. An activation function layer applies the necessary non-linearity, while a dropout layer helps prevent overfitting. The model concludes with a dense output layer of 5 units, with 1,285 parameters, providing the final classification output.

#### 3.6.3 InceptionV3

It is an improvised version of the basic model of Inception which has 42 layers as well as a low error rate than its predecessors (28). Inception module is the primary characteristic of the InceptionV3 architecture which consists of convolutional layers of various types and sizes. The work of the Inception module is to enable the model in learning a wide variety of feature representations at various levels of abstraction without the requirement of any substantial increase in the number of parameters. Batch normalization and dropout regularization techniques are also used to prevent the overfitting error and improve the performance of the model which is an essential aspect of the InceptionV3 architecture. In general, the InceptionV3 architecture is a robust as well as adaptable deep learning model that can be applied to a variety of image recognition tasks. Mathematically, it can be shown by Equation 23:


(23) Inception(x)= Concatenate(tower1(x), tower 2(x),tower 3(x), tower 4(x)) 

Each tower in the concatenation is composed of different layers for pooling and convolution, each with its own particular kernel sizes. The purpose of these towers is to record various levels of spatial hierarchies present in the input data. **Table 7** presents the layered architecture of InceptionV3 and showcases the number of parameters of each layer.

**Table 7:** Layered architecture of InceptionV3.

Layers	Output Shape	Parameters
**InceptionV3** **Globalaveragepooling2d** Dense layer Batch normalization Activation function Dropout Dense layer	(None,5,5,1536) (None,1536) (None,256) (None,256) (None,256) (None,256) (None,5)	54386756 0 493216 1024 0 0 1285

The architecture begins with the InceptionV3 base model, which outputs a tensor with the shape (None, 5, 5, 1536) and includes a substantial 54,386,756 parameters. This base model is highly capable of capturing complex and detailed features from input images due to its sophisticated inception modules. Following this, a Global Average Pooling 2D layer is used to reduce the spatial dimensions of the output tensor, resulting in a shape of (None, 1536). This layer does not introduce any additional parameters but serves to summarize the spatial information across the feature maps. Next, a dense layer with 256 units is added, involving 493,216 parameters. This layer enables the network to learn and represent higher-level features from the pooled features. To ensure training stability and improve convergence, a batch normalization layer is included, adding 1,024 parameters. This layer normalizes the inputs to the subsequent activation function, which introduces non-linearity without increasing the parameter count with a dropout layer as zero. The architecture concludes with a dense output layer comprising 5 units, which corresponds to the number of classes in the classification task. This final layer includes 1,285 parameters.

#### 3.6.4 Densenet169

DenseNet, short for Densely Connected Convolutional Networks, is a type of CNN architecture. This architecture is designed to address issues like the vanishing gradient problem by connecting each layer to every other layer in a dense manner. One distinctive feature of DenseNet is its use of dense blocks, where every layer receives input from all preceding layers in the block. This dense connectivity helps in feature reuse and encourages the flow of gradients throughout the network during backpropagation. This can be especially beneficial in training deep networks. In the case of DenseNet169, the model has a total of 169 layers, and the last fully connected layer has been omitted. Instead, there are three fully connected layers with 256, 128, and 10 nodes respectively (29). The softmax activation function is applied to the last layer, which is a common choice for multi-class classification problems. Additionally, batch normalization and a 40% dropout are applied to the fully connected layers. Batch normalization helps in normalizing the inputs to a layer, which can speed up training and improve generalization. Mathematically, the output of the l-th layer in DenseNet is computed using the Equation 24:


(24) $$\;\;\;\;\;\;\;\;\;\;\;\;\;\;H_{l}\left(X_{l}\right)=\;H_{l-1}\left(\left[X_{0},X_{1},X_{2},\ldots \ldots .,X_{l-1}\right]\right)$$

Here, X_0_ = input image, $H_{l}\left(X_{l}\right)$ = feature maps, and [] = concatenation operation. **Table 8** presents the layered architecture of DenseNet169 and showcases the number of parameters of each layer.

**Table 8:** Layered architecture of DenseNet169.

Layers	Output Shape	Parameters
**DenseNet169** **Globalaveragepooling2d** Dense layer Batch normalization Activation function Dropout Dense layer	(None,7,7,2048) (None,2048) (None,256) (None,256) (None,256) (None,256) (None,5)	58269848 0 559288 1024 0 0 1285

The DenseNet169 architecture starts with the DenseNet169 base, producing an output tensor with the shape (None, 7, 7, 2048) and containing 58,269,848 parameters. This base model is composed of dense blocks and transition layers that ensure efficient feature reuse and network depth, allowing it to capture detailed and hierarchical features from input images. Following the DenseNet169 layer, a Global Average Pooling 2D layer is applied, which reduces the spatial dimensions to (None, 2048) by averaging each feature map. This layer does not add any parameters but effectively summarizes the spatial information from the previous layer. Subsequently, a dense layer with 256 units is introduced, involving 559,288 parameters. This layer serves to further process and condense the features extracted by the base model, enhancing the ability of the network to learn difficult patterns. A batch normalization layer follows, adding 1,024 parameters to normalize the outputs of the dense layer. This normalization improves training stability and accelerates convergence by standardizing the inputs to the activation function. An activation function layer then applies a non-linear transformation to the normalized outputs, enhancing the model’s capacity to capture intricate relationships in the data without increasing the parameter count. Finally, the architecture concludes with a dense output layer comprising 5 units, corresponding to the number of target classes, and introducing 1,285 parameters.

### 3.7 Evaluative parameters

When applying deep learning techniques to gastric cancer detection, various evaluation metrics are crucial to comprehensively assess model performance (30–32). Accuracy measures the proportion of correctly predicted cases (both true positives and true negatives) against the total number of cases. It provides an overall indication of the model’s ability to make correct predictions. However, accuracy alone may not be sufficient, especially in imbalanced datasets where the number of negative cases far exceeds positive ones. Loss quantifies the error in the model’s predictions by calculating the average squared difference between the actual and predicted values. A lower loss indicates a better fit of the model to the training data. Root Mean Square Error (RMSE) also evaluates prediction quality but emphasizes larger errors more than smaller ones, as it takes the square root of the mean squared error. In the context of gastric cancer detection, a lower RMSE would indicate that the model’s predictions are closer to the actual cancer diagnoses, enhancing its reliability. Precision focuses on the accuracy of the positive predictions made by the model. It is particularly important in medical diagnostics to minimize the number of false positives, ensuring that patients are not incorrectly diagnosed with cancer. Recall, on the other hand, measures the model’s ability to correctly identify actual positive cases (i.e., true cancer cases). High recall is critical in gastric cancer detection to ensure that as many true cancer cases as possible are identified, reducing the risk of missed diagnoses. F1 score combines both precision and recall into a single metric, providing a balanced measure of the model’s performance. This is especially useful when there is a need to find a compromise between precision and recall, ensuring that the model not only identifies most of the cancer cases but also maintains a high accuracy of positive predictions. Together, these metrics provide a comprehensive evaluation framework, ensuring that deep learning models for gastric cancer detection are both accurate and reliable in clinical applications.


(25) $$Accuracy=\;\frac{True\;Positive\;+\;True\;Negative}{True\;Positive\;+\;True\;Negative\;+False\;Positive\;+False\;Negative}$$

(26) $$Loss=\;\frac{{\left(Actual\;Value-Predicted\;Value\right)}^{2}}{Number\;of\;Observatiosn}$$

(27) $$RMSE=\;\sqrt{\frac{{\left(Actual\;Value-Predicted\;Value\right)}^{2}}{Number\;of\;Observatiosn}}$$

(28) $$Precision=\;\frac{True\;Positive}{True\;Positive\;+False\;Positive\;}$$

(29) $$Recall=\;\frac{True\;Positive}{True\;Positive+False\;Negative}$$

(30) $$F1\;score=2\frac{Precision*Recall}{Recall+Precision}$$

## 4 Results

The results of applied deep learning models like NASNetMobile, InceptionV3, EfficientnetB5, EfficientNetB6, DenseNet169, and the hybrid model (EfficientNetB6 and DenseNet169) for various gastric diseases is being presented. We have discussed the accuracy, loss, and RMSE of the models at the training and testing phase for the whole dataset along with their graphical analysis. In addition, the models have also been evaluated for the various classes of the dataset using the same parameters indicated in Section 3. At the end of this section, we have discussed the overall results of their research and compared them with the existing techniques.

**Table 9** presents the performance metrics of various Convolutional Neural Networks (CNNs) and a hybrid model comprising EfficientNetB6 and DenseNet169 based on their accuracy along with the loss values for both the training and testing phases.

**Table 9:** Evaluation of classifiers for training and testing phase.

Classifiers	Training		Testing	
	Accuracy	Loss	Accuracy	Loss
**NASNetMobile**	96.56	0.086	95.28	0.359
**EfficientNetB5**	99.59	0.159	98.40	0.159
**EfficientNetB6**	92.86	0.059	99.88	0.049
**InceptionV3**	97.59	0.056	98.32	0.186
**DenseNet169**	99.56	0.053	96.88	0.259
**Hybrid Model (EfficientNetB6 + DenseNet169)**	98.59	0.059	99.6	0.0896

EfficientNetB6 stands out with an impressive testing accuracy of 99.88% and a remarkably low testing loss of 0.049, indicating its strong generalization capability despite a lower training accuracy of 92.86%. This model’s low testing loss suggests that it effectively minimizes errors on unseen data. EfficientNetB5 also demonstrates high performance, with a training accuracy of 99.59% and a consistent testing accuracy of 98.40%, coupled with an identical training and testing loss of 0.159. InceptionV3 and DenseNet169 both exhibit high training accuracies of 97.59% and 99.56%, respectively, but their testing accuracies of 98.32% and 96.88% suggest a slight overfitting, as indicated by their higher testing losses of 0.186 and 0.259. NASNetMobile, while achieving a respectable training accuracy of 96.56%, shows a notable drop in testing accuracy to 95.28% and a higher testing loss of 0.359, indicating less robustness in handling new data. The hybrid model, combining EfficientNetB6 and DenseNet169, achieves a strong performance with a testing accuracy of 99.6% and a relatively low testing loss of 0.0896, highlighting the potential benefits of integrating complementary models to enhance overall predictive power. These results underscore the importance of balancing high accuracy with low loss to ensure robust and reliable model performance in practical applications.

**Figure 6:**
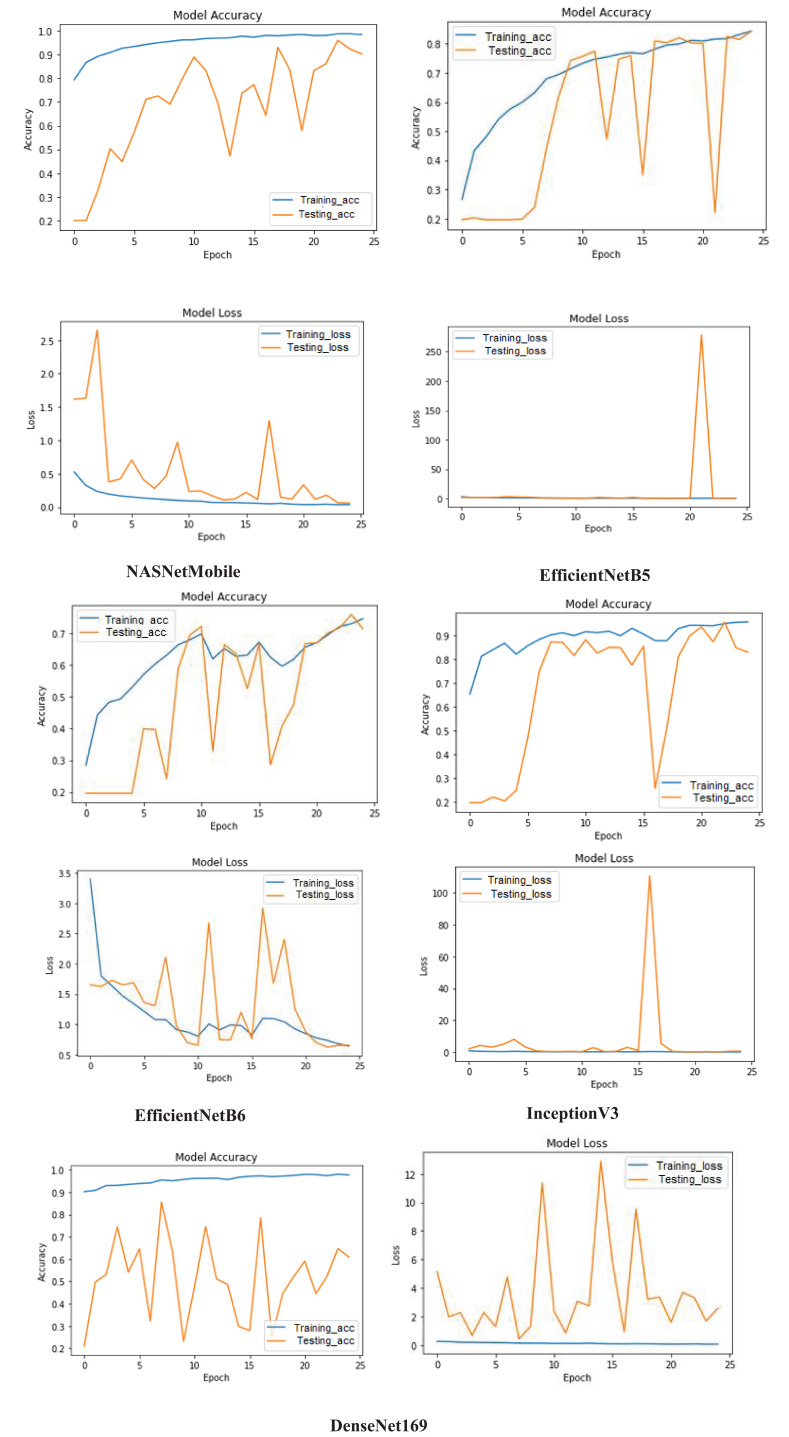
Performance analysis of models on the basis of their learning curves.

In addition to this, the training (blue) and testing (orange) accuracy as well as loss learning curves for the models is depicted in **Figure 6** to provide a comprehensive overview of their performance over 25 epochs. The *NASNetMobile* steadily increases the training accuracy and stabilizes around 0.96 which demonstrates its effective learning from the training data. However, the testing accuracy identifies overfitting despite of reaching to high accuracy because of its significant fluctuations. This is further supported by the loss graph, where the training loss consistently decreases and reflects minimum errors on training data. In contrast, the testing loss exhibits substantial spikes and variability, particularly early in the training process, which underscores the instability in the model’s performance on unseen data. In case of *EfficientNetB5*, the accuracy graph displays a smooth and consistent increase in training accuracy which indicates effective learning from the training data. However, the testing accuracy shows extreme fluctuations, with sharp drops to zero and sudden recoveries. The loss graph further emphasizes this issue; while the training loss remains consistently low, the testing loss spikes dramatically at around epoch 20, suggesting a severe overfitting problem where the model performs well on training data but poorly on testing data. For *EfficientNetB6*, the accuracy graph shows that training accuracy steadily increases and reaches around 0.75, indicating the model’s capability to learn from the training data. However, the testing accuracy is highly erratic, with frequent sharp declines and recoveries. The loss graph complements this observation, with the training loss gradually decreasing while as the testing loss (displays significant fluctuations, particularly with notable spikes, highlighting inconsistencies in the model’s performance on testing data. For *InceptionV3*, the accuracy graph shows the training accuracy steadily increasing and stabilizing around 0.95, indicating effective learning from the training data. The testing accuracy also shows an initial rise, stabilizing around epoch 5 to values close to the training accuracy, indicating good generalization during the earlier epochs. However, there is a notable drop around epoch 15, suggesting a transient issue before recovery. The loss graph further elucidates these trends, with the training loss consistently decreasing and remaining low but the testing loss shows fluctuations, particularly a significant spike around epoch 15, corresponding to the drop in testing accuracy, indicating an overfitting issue or instability during that period. For *DenseNet169*, the accuracy graph demonstrates a high and stable training accuracy close to 0.98, indicating that the model learns effectively from the training data. However, the testing accuracy fluctuates significantly, with sharp rises and falls throughout the epochs, suggesting instability and poor generalization to unseen data. Similarly, the loss graph shows the training loss remaining consistently low, confirming that the model minimizes errors on the training data effectively. In contrast, the testing loss exhibits substantial volatility with frequent spikes, indicating inconsistent performance on the testing set.

In addition, **Table 10** presents the performance analysis of various models based on their RMSE values during both training and testing phases.

**Table 10:** Analyzing models based on their RMSE values.

Models	Training	Testing
**NASNetMobile**	0.35	0.76
**EfficientNetB5**	0.64	0.62
**EfficientNetB6**	0.37	0.32
**InceptionV3**	0.28	0.44
**DenseNet169**	0.39	0.57
**Hybrid Model (EfficientNetB6 + DenseNet169)**	0.55	0.48

Among the individual models, InceptionV3 exhibited the lowest RMSE values during training (0.28) and testing (0.44), which indicates its superior performance in capturing the underlying patterns in the data. EfficientNetB6 also demonstrated strong performance with low RMSE values of 0.37 in training and 0.32 in testing. EfficientNetB5, while having a slightly higher RMSE in training (0.64), displayed a competitive performance with an RMSE of 0.62 in testing. NASNetMobile showed a notable difference between training (0.35) and testing (0.76) RMSE values, suggesting a potential risk of overfitting. DenseNet169 presented balanced performance with RMSE values of 0.39 in training and 0.57 in testing. The hybrid model, combining EfficientNetB6 and DenseNet169, showcased a trade-off, achieving an RMSE of 0.55 in training and 0.48 in testing. Overall, these results generate insights into the relative strengths and weaknesses of each model, guiding the selection of the model which is the most suitable on the basis of specific requirements of the task at hand. Likewise, the models have been also examined for the single dataset on the basis of different parameters in **Table 11**.

**Table 11:** Comparing the performance of multiple learning models.

Models	Precision	Recall	F1 score
**NASNetMobile**	87.8	93.76	90.78
**EfficientNetB5**	95.16	95.18	92.18
**EfficientNetB6**	97.94	97.34	96.16
**InceptionV3**	94.2	95.96	95.4
**DenseNet169**	95	95.58	92.6
**Hybrid Model (EfficientNetB6 + DenseNet169)**	92.4	93.16	89.98

Starting with NASNetMobile, it achieves a precision of 87.8% which indicates that 87.8% of the instances predicted as positive were actually positive. The recall is 93.76%, indicating that it successfully identified 93.76% of the actual positive instances. The F1 score, which considers both precision and recall, is 90.78%. EfficientNetB5 demonstrates a higher precision of 95.16%, suggesting a better ability to accurately predict positive instances. The recall is 95.18%, indicating that it captured a high proportion of actual positive instances with 92.18% of F1 score is 92.18. Likewise, other models such as EfficientNetB6 exhibits even higher precision at 97.94%, recall of 97.34%, and F1 score of 96.16% which demonstrates an overall a good performance. InceptionV3 and DenseNet169 also computed 94.2% and 95% as precision score, 95.96% and 95.58% as recall, and 95.4% and 92.6% as F1 score respectively which reflects a balanced performance.

The Hybrid Model, which combines EfficientNetB6 and DenseNet169, achieves a precision of 92.4%, suggesting a slightly lower accuracy in predicting positive instances compared to other models. The recall is 93.16%, indicating a good ability to capture actual positive instances. The F1 score is 89.98%, indicating a slightly lower overall performance compared to individual models. In a nutshell, EfficientNetB6 consistently demonstrates high precision and recall, resulting in a strong F1 score. The Hybrid Model shows a slightly lower F1 score and precision but still maintains a good overall performance.

**Figure 7:**
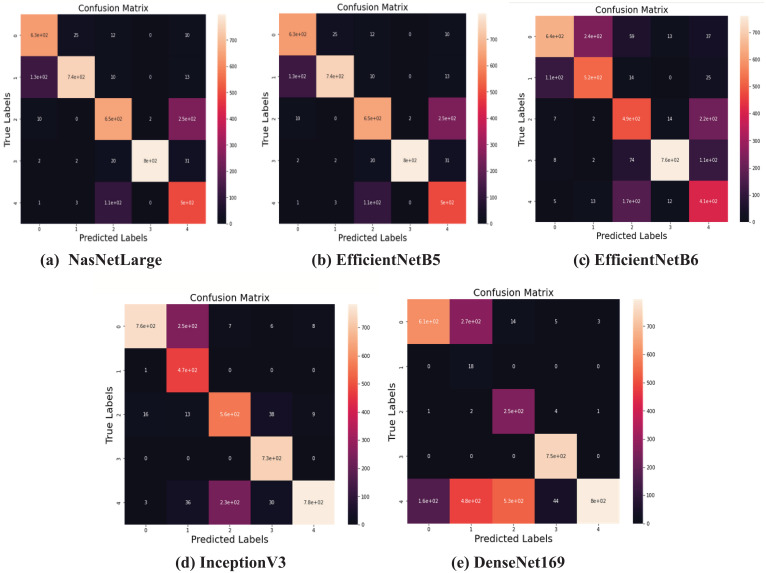
Confusion Matrix of the applied models. **(A)** NasNetLarge; **(B)** EfficientNetB5; **(C)** EfficientNetB6; **(D)** InceptionV3; **(E)** DenseNet169.

Apart from this, as shown in **Figure 7**, the confusion matrix has been also generated for comparing the efficacy of the deep transfer learning models by calculating the actual and predicted rates for each class of gastrointestinal disorders. Accuracy, precision, and other evaluative parameters have been estimated based on this confusion matrix.

After examining the models for the combined dataset, they have been now evaluated to test how they perform for different classes of the applied dataset i.e. gastric cancer diseases on the basis of same parameters as shown in **Table 12**.

**Table 12:** Examining the results of models for multi-classes of dataset.

Gastric cancer	Models	Training			Testing		
		Accuracy	Loss	RMSE	Accuracy	Loss	RMSE
Normal Pylorus	**NASNetMobile**	98.88	0.151	0.561	93.86	0.471	0.273
	**EfficientNetB5**	95.81	0.285	0.450	95.45	0.295	0.941
	**EfficientNetB6**	96.94	0.595	0.628	95.85	0.176	0.159
	**InceptionV3**	99.43	0.558	0.450	97.94	0.246	0.146
	**DenseNet169**	97.69	0.695	0.258	90.45	0.185	0.862
	**Hybrid Model (EfficientNetB6 + DenseNet169)**	86.80	0.151	0.522	95.56	0.175	0.963
Normal Z-line	**NASNetMobile**	95.45	0.599	0.275	98.75	0.864	0.232
	**EfficientNetB5**	95.75	0.755	0.941	94.95	0.596	0.475
	**EfficientNetB6**	96.91	0.536	0.159	97.85	0.750	0.295
	**InceptionV3**	96.45	0.590	0.146	96.34	0.587	0.334
	**DenseNet169**	91.35	0.759	0.862679	91.49	0.861	0.500
	**Hybrid Model (EfficientNetB6 + DenseNet169)**	99.75	0.590	0.963	96.97	0.598	0.133
Polyps	**NASNetMobile**	99.75	0.953	0.249	86.28	0.594	0.679
	**EfficientNetB5**	94.59	0.356	0.612	98.40	0.576	0.585
	**EfficientNetB6**	99.56	0.259	0.488	97.88	0.560	0.426
	**InceptionV3**	93.48	0.256	0.577	91.32	0.467	0.785
	**DenseNet169**	91.58	0.200	0.451	90.88	0.481	0.559
	**Hybrid Model(EfficientNetB6 + DenseNet169)**	99.68	0.234	0.598	90.86	0.868	0.864
Stool	**NASNetMobile**	98.59	0.086	0.286	95.48	0.386	0.621
	**EfficientNetB5**	93.75	0.456	0.486	96.86	0.176	0.419
	**EfficientNetB6**	98.59	0.085	0.284	92.16	0.096	0.309
	**InceptionV3**	98.56	0.759	0.275	95.86	0.149	0.386
	**DenseNet169**	97.86	0.856	0.259	90.46	0.246	0.495
	**Hybrid Model (EfficientNetB6 + DenseNet169)**	98.78	0.095	0.156	96.59	0.086	0.293
Ulcerative Colitis	**NASNetMobile**	98.86	0.753	0.175	75.28	1.304	1.551
	**EfficientNetB5**	91.46	0.955	0.495	59.40	2.146	1.382
	**EfficientNetB6**	96.24	0.865	0.148	98.88	0.050	0.223
	**InceptionV3**	94.26	0.468	0.136	86.32	0.127	0.356
	**DenseNet169**	99.56	0.865	0.286	97.88	0.231	0.480
	**Hybrid Model (EfficientNetB6 + DenseNet169)**	99.75	0.957	0.954	97.6	0.088	0.296

For normal pylorus, Inception v3 obtained the best accuracy by 95.43% and 97.94%, respectively, while hybridization of EfficientNetB6 and DenseNet169 obtained the best training and testing loss by 0.151 and 0.175. For Normal Z-line, the hybrid model and InceptionV3 obtained the best training accuracy of 99.75% and loss of 0.87. For the best accuracy and loss, EfficientNetB6 stood at the top by 97.85% and 0.536, respectively. For polyps, NASNetMobile and EfficientNetB5 obtained the highest training and testing accuracies with 99.75% and 98.40%, respectively. On the other hand, the best loss has been achieved by DenseNet169 and InceptionV3 by 0.200 and 0.467. For stool, EfficientNetB5 and B6 obtained the best accuracy along with a loss of 96.86% and 0.085, respectively. For the best training accuracy and loss, the hybrid model stood at the top by 98.78% and 0.086, respectively. In the end, for ulcerative colitis, densenet169 and efficientNetB6 obtained the highest training and testing accuracy at 99.56% and 98.88%, respectively. In contrast, the best training and testing loss value has been obtained by InceptionV3 and EfficientNetB6 by 0.468 and 0.050, respectively.

Similar to **Table 11**, the performance of the models have been again assessed but for different classes of dataset using the parameters as shown in **Table 13**.

**Table 13:** Classification of diseases by analyzing the performance of models.

Models	Classes	Precision	Recall	F1-Score
**NASNetMobile**	**Normal Pylorous**	99.0	85.0	87.0
	**Normal z line**	59.0	92.0	73.0
	**Polyps**	86.0	99.9	99.9
	**Stool**	96.0	92.0	95.0
	**Ulcerative Colitis**	99.0	99.9	99.0
**EfficientNetB5**	**Normal Pylorous**	85.0	97.0	86.0
	**Normal z line**	99.0	89.0	87.0
	**Polyps**	92.9	98.9	95.9
	**Stool**	99.0	92.0	99.0
	**Ulcerative Colitis**	99.9	99.0	93.0
**EfficientNetB6**	**Normal Pylorous**	97.0	93.0	99.0
	**Normal z line**	99.9	99.0	93.0
	**Polyps**	98.9	96.9	96.9
	**Stool**	95.0	99.9	96.0
	**Ulcerative Colitis**	98.9	97.9	95.9
**InceptionV3**	**Normal Pylorous**	92.0	87.0	92.0
	**Normal z line**	89.0	97.0	92.0
	**Polyps**	98.0	98.9	99.0
	**Stool**	96.0	99.0	98.0
	**Ulcerative Colitis**	96.0	97.9	96.0
**DenseNet169**	**Normal Pylorous**	88.0	99.0	86.0
	**Normal z line**	99.0	88.0	86.0
	**Ulcerative Colitis**	97.0	99.0	99.0
	**Polyps**	92.0	93.9	99.0
	**Stool**	99.0	98.0	93.0
**Hybrid Model(EfficientNetB6 + DenseNet169)**	**Normal Pylorous**	96.0	95.0	93.0
	**Normal z line**	89.0	83.0	98.0
	**Stool**	97.0	96.0	86.0
	**Polyps**	92.0	97.9	96.9
	**Ulcerative Colitis**	88.0	93.9	76.0

As shown in the table, NASNetMobile computed the highest precision of 99% for normal pylorus and ulcerative colitis, recall and an F1 score of 99% for polyps and ulcerative polyps. The model has obtained the least value of precision, recall, and F1 score for the normal z line by 59%, 85%, and 73%. EfficientNetB5 computed the highest precision and recall of 99.9% for ulcerative colitis and an F1 score of 99% for stool. The model has obtained the least value of precision, F1 score, and recall for normal pylorus by 85% and 86%, respectively, and normal z line by 89%. EfficientNetB6 computed the highest precision of 99% for normal z line, recall of 99% for normal z line and stool, and F1 score of 99% for normal pylorus. The model has obtained the least value of precision, recall, and F1 score for stool, normal pylorus, and normal z line by 95%, 93%, and 93%.

InceptionV3 computed the highest precision of 99% for polyps, recall of 99% for stool, and F1 score (99%) for polyps. The model has computed the least value of precision, recall, as well as F1 score for normal pylorus line with 92%, 87%, and 92% respectively. DenseNet169 computed the highest precision of 99% for normal z line and stool, recall of 99% for normal pylorus and ulcerative polyps, and F1 score of 99% for polyps and ulcerative polyps. The model has obtained the least value of precision and recall for normal pylorus by 88% each and F1 score for normal z line by 86%. The hybrid model computed the highest precision of 97% for stool, recall of 97.9% for polyps, and F1 score of 98% for normal z line. The model has obtained the least value of precision, recall, and F1 score for the normal z line by 89%, 83%, and 86%, respectively.

**Figure 8:**
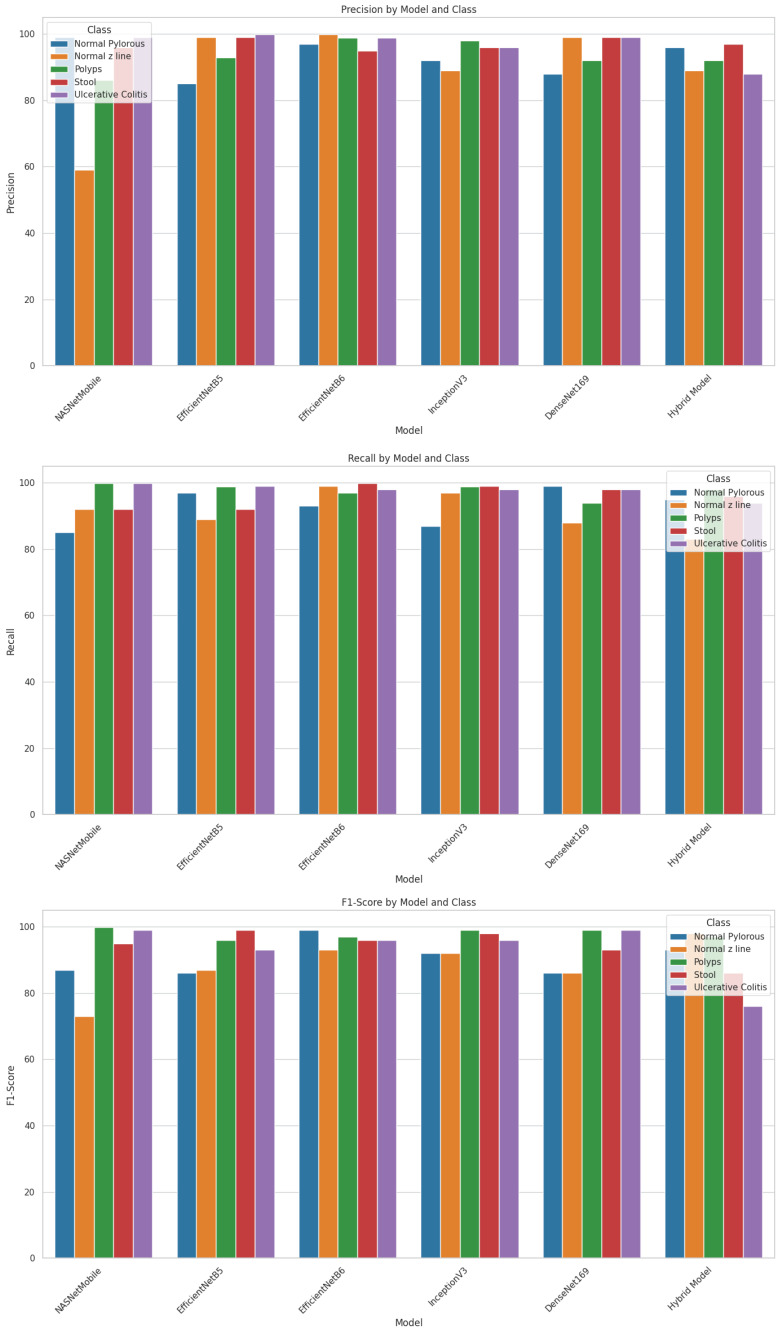
Evaluation of models using gastric cancer dataset.

These values of the models are also represented in the form of bar graph so that they can be compared and make it easy to identify which model performs better for a particular metric as shown in **Figure 8**.

In addition, the proposed automated system has been contrasted to existing techniques based on their datasets, methods, and accuracy, as shown in **Table 14**.

**Table 14:** Analysis of current work with the existing one.

Author’s Name	Dataset	Technique	Accuracy
Li et al. (2018) (12)	560 gastric slices, 140 normal slices	Gastric Net	97.93%
Song et al. (2020) (14)	PLAGH dataset	DeepLabV3	80.6%
Zhou et al. (2014) (19)	359 VCE frames	SVM classifier	90.77%
Sun et al. (2020) (24)	Annotated gastricscopic image dataset	Densely connected neural network	96.79%
Yong et al. (2023) (36)	Gastric Histopathology Sub-size Image	Ensemble deep learning methods	99.20%
Asperti & Mastronardo (2017) (22)	Images taken from Kvasir dataset	Inception	91.55%
Zhang et al. (2024) (32)		Improved Mask R-CNN	93.9%
Naz et al. (2021) (33)		Ensemble Learning Classifier	86.4%
Thomas et al. (2023) (34)		EfficientNetB0	98.01%
Khan et al. (2022) (35)		MobileNetV2+Baysian Optimization	98.20%
**Our study**		**Hybrid (EfficientNetB6+ DensenNet169)**	**99.75%**

The bold value represents the best value computed by the model in this paper.

Initially for the different dataset of gastric cancer images, it has been found that Li et al. (2018) achieved a high accuracy of 97.93% using Gastric Net on a dataset of 560 gastric slices and 140 normal slices. Likewise, Song et al. (2020) utilized DeepLabV3 on the PLAGH dataset, resulting in a lower accuracy of 80.6%. Zhou et al. (2014) employed an SVM classifier on 359 VCE frames, achieving 90.77% accuracy. Sun et al. (2020) used a densely connected neural network on an annotated gastroscopic image dataset, yielding an accuracy of 96.79% while as Yong et al. (2023) obtained 99.20% accuracy on Gastric Histopathology Sub-size Image database using ensemble deep learning techniques.

On the other hand for the Kvasir dataset which has also been used in this paper, it has been discovered that Asperti and Mastronardo (2017) obtained an accuracy of 91.55% with the Inception model. Zhang et al. (2024) reported a 93.9% accuracy using an improved Mask R-CNN, while Naz et al. (2021) achieved 86.4% accuracy with an ensemble learning classifier. Thomas et al. (2023) and Khan et al. (2022) achieved high accuracies of 98.01% and 98.20%, respectively, using EfficientNetB0 and a combination of MobileNetV2 with Bayesian optimization. However, our study surpasses all these approaches, attaining the highest accuracy of 99.75% using a hybrid model that integrates EfficientNetB6 and DenseNet169. This comparative analysis underscores the effectiveness of hybrid models in achieving superior classification performance in medical image analysis.

## 5 Conclusion

The research aimed at the detection and classification of various gastric diseases which include ulcerative colitis, normal pylorus, polyps, normal-Z line, and stool, using endoscopic images. Various advanced deep learning models had been used and trained using this dataset. During experimentation, it had been seen that Inception-V3 demonstrated the highest testing accuracy of 97.94% for normal pylorus, while EfficientNetB6 outperformed others with a testing accuracy of 97.85% for normal-Z line. Besides this, EfficientNetB5 excelled in detecting polyps, achieving the highest testing accuracy of 98.40%, and also led in stool classification with a testing accuracy of 96.86%. Notably, EfficientNetB6 achieved the highest testing accuracy for ulcerative colitis at 98.88%. Irrespective of this, certain challenges had been also found such as challenges such as the variations in the size of image and the presence of black borders which impacted the overall accuracy of gastric disease detection and classification. Thus, to address these challenges, our study suggests potential avenues for improvement. Future work should focus to enhance the quality of images by applying advanced image processing technologies. This will be crucial to optimize the performance of gastric disease detection and classification. Moreover, the developed model can also be deployed at medical centers so that the end users through an application could empower patients in identifying their gastro-intestinal disorders promptly. This application will also serve as a valuable tool in facilitating early diagnosis and intervention.

## Data Availability

Publicly available datasets were analyzed in this study. This data can be found here: https://dl.acm.org/do/10.1145/3193289/full/.
